# Dual role of rVSV vectors in prophylactic and therapeutic innovation: a comprehensive bibliometric and clinical trial perspective

**DOI:** 10.3389/fimmu.2026.1711059

**Published:** 2026-02-16

**Authors:** Yueqing Chen, Yongxian Zhang, Yifei Zhang, Changwen Bie, Runze Wang, Yan Zhao, Mijia Lu

**Affiliations:** 1 School of Laboratory Medicine and Bioengineering, Hangzhou Medical College, Hangzhou, Zhejiang, China; 2 Department of Microbiology, Perelman School of Medicine, University of Pennsylvania, Philadelphia, PA, United States; 3 College of Food Science and Nutritional Engineering, China Agricultural University, Beijing, China; 4 Department of Population Health Science, Weill Cornell Medicine, Cornell University, New York, NY, United States; 5 Department of Emergency Medicine, The Fourth Affiliated Hospital of Zhejiang University, School of Medicine, Yiwu, Zhejiang, China

**Keywords:** bibliometric analysis, CiteSpace, clinical trial, immunotherapy, oncolytic, recombinant vesicular stomatitis virus, vector vaccine

## Abstract

**Background:**

Recombinant Vesicular Stomatitis Virus (rVSV) vector vaccines have become essential in combating emerging viral diseases and have shown promise in immunotherapy, particularly in the discipline of cancer treatment. Although extensive research has been conducted in this rVSV field, a comprehensive bibliometric and landscape analysis is lacking.

**Objective:**

To depict the current research landscape of recombinant vaccines and antitumor agents based on the VSV vector, especially of the critical focus areas, emerging trends, and ongoing clinical trials.

**Methods:**

The software CiteSpace was used to conduct the bibliometric analysis to visualize rVSV research trends, prominent authors, leading institutions, contributing countries, frequently used keywords, and the top 10 most cited articles. Additionally, the advancements and research directions were explored by analyzing active clinical trials related to the rVSV field.

**Results:**

A total of 311 publications from 1996 to 2024 were screened out from the Web of Science Core Collection based on predefined inclusion criteria. The cumulative annual publication volume showed a steady increase, with the United States and Canada leading in research output. Notable authors such as Heinz Feldmann and John K. Rose were identified as the most frequently cited contributors. Key cluster and reference analyses revealed a shift from studying basic mechanisms to focusing on vaccine construction and clinical trials. Furthermore, efficient trials were identified in both infectious disease vaccines and antitumor agents, providing insights into potential applications in the indication expansion of currently applied vaccines and precise targeting strategies in newly developed tumor agents.

**Conclusion:**

Benefited by the CiteSpace-generated bibliometric analysis, this study mapped the evolution of research and highlighted key topics in rVSV vaccine development. The comprehensive review of recent advancements and active clinical trials (using clinicaltrial.gov) offers valuable insights into current trends and future research directions.

## 1 Introduction

The Vesicular Stomatitis Virus (VSV) belongs to the Vesiculovirus genus of the Rhabdoviridae family and primarily affects livestock such as cattle, horses, and swine, with dominating variants including VSIV, VSNJV, and COCV, with most recombinant VSV (rVSV) vaccines using the VSIV strain (1). The VSV genome consists of a non-segmented 11KB negative-sense RNA that encodes five structural proteins: the nucleocapsid (N), polymerase (L), phosphoprotein (P), glycoprotein (G), and matrix protein (M). As an enveloped virus, VSV features trimeric glycoproteins, classified as type 1 membrane proteins in trimeric polypeptides (2) and functionally categorized as a Class III fusion protein due to its complex fusion mechanism (3). The G protein, VSV’s key surface antigen, facilitates viral entry via low-density lipoprotein (LDL) receptors across various mammalian and insect cells and serves as the primary target for neutralizing antibodies (4).

The structural simplicity of VSV enables efficient genomic modifications, facilitating the development of reverse genetics systems that have significantly expanded VSV’s research applications, particularly in vaccine design (5). rVSV is typically engineered by replacing its native G protein with glycoproteins (GP) from other viruses, thereby altering its tropism to target specific cell types for gene delivery or vaccine development. Alternatively, some rVSV constructs retain the native G protein while incorporating additional viral antigenic protein to elicit immune responses against the source virus. This adaptability makes rVSV a versatile platform for developing vaccines, therapeutic gene delivery systems, and oncolytic viruses targeting cancer cells selectively (6, 7). The part of the bibliometric analysis investigated the evolution of research themes, collaboration networks, and key trends in VSV-based vaccine development, and underscored the strategic role in advancing innovative and effective medical interventions.

## 2 Materials and methods

### 2.1 Data acquisition

Literature identification was conducted using structured bibliographic databases (Web of Science Core Collection) and a clinical trial registry (ClinicalTrials.gov), in accordance with the PRISMA 2020 guidelines for new systematic reviews based on database and register searches only.

The Web of Science Core Collection (WoSCC) was selected as the primary source for visual data acquisition due to its extensive coverage of academic literature across a wide range of disciplines. This database encompasses approximately 34,000 journals, making it a comprehensive resource for bibliometric analysis (8). The search focused on publications related to rVSV vector constructs, either for disease prevention or treatment, covering the period from 1996 to 2024. The bibliometric search conducted on October 4, 2024, yielded 1,396 relevant records, following the search strategy detailed in **Table 1**.

**Table 1:** Search steps.

Search steps	Search terms
#1	Vesicular stomatitis virus (Topic) OR VSV (Topic) OR Vesicular stomatitis Indiana virus (Topic) OR Vesicular stomatitis New Jersey virus (Topic) OR Recombinant vesicular stomatitis virus (Topic) OR rVSV (Topic) AND Preprint Citation Index (Exclude – Database)
#2	Vaccine (Topic) OR Vaccines (Topic) OR Vector (Topic) OR Oncolytic (Topic) AND Preprint Citation Index (Exclude – Database)
#3	Combination of #1 AND #2

Although the EU Clinical Trials Register (EudraCT) and ChiCTR may also contain relevant trials, the clinical trial database (https://clinicaltrials.gov/) was utilized to identify efficient rVSV vector-related trials due to its greater accessibility, standardized query interface, and inclusion of multinational trials sponsored by global organizations and pharmaceutical companies. Active trials were identified and manually screened with “primary completion date” on or after January 1, 2019. Those with study type “intervention”(rather than observation) and status labelled “Completed” trials were included; For those labelled “withdrawn”, “terminated”, or “Unknown”, items were excluded. The search terms, inclusion criteria, and findings are presented in Section 3. Different search terms were applied to categorize two groups: Prevention of emerging infectious diseases and antitumor viro-immunotherapy.

Search Date: Started on October 4, 2024
Search Terms (WoS): Vesicular stomatitis virus, VSV, Vesicular stomatitis Indiana virus, Vesicular stomatitis New Jersey virus, Recombinant vesicular stomatitis virus, rVSV, Vaccine, Vaccines, Vector vaccine, Vector vaccines

Inclusion criteria:

- Research Subject: Studies focusing on Recombinant Vesicular Stomatitis Virus Vector Vaccines;
- Intervention: Research centered on the construction and application of rVSV vaccine.

Exclusion criteria:

- Studies not directly related on recombinant VSV vector vaccines.
- Research not involving the construction and application of these vaccines.
- Document types such as abstracts, editorial materials, letters, and news reports.

Data were extracted and formatted into text files, which included comprehensive details such as the author, institution, country, abstract, keywords, publication date, and cited references.

### 2.2 Data analysis

For this study, we employed the CiteSpace software (version 2.6.2) (9) to conduct a comprehensive visualization analysis of the scientific literature collected from WOSCC, spanning the years 1996 to 2024. The time slicing was configured at intervals of either two or four years, based on the density and distribution of publications over the selected timeframe, to ensure streamlined data extraction for analysis. Unless otherwise specified, default parameter settings were applied for all analyses.

CiteSpace Software Configuration

- Selection method: Pathfinder, pruning of sliced networks.
- Time slicing: 1996 to 2024, with each time slice set to either 2 or 4 years.
- Other parameters: default setting applied.

Scope of Visualization Analysis: The analysis focused on visualizing the relationships among key research entities, including:

- Authors: Identifying prolific contributors in the field.
- Institutions: Mapping collaboration networks and leading research centers.
- Countries: Highlighting geographic distribution of research efforts.
- Keywords: Analyzing prevalent themes and emerging trends in rVSV vaccine research.
- References: Visualize the information involving with the top-most cited or strongest burst references.

These analyses provided insights into the collaborative landscape and thematic evolution within the domain of recombinant VSV vector-related vaccines research.

## 3 Results

### 3.1 Inclusion of literature

**Figure 1:**
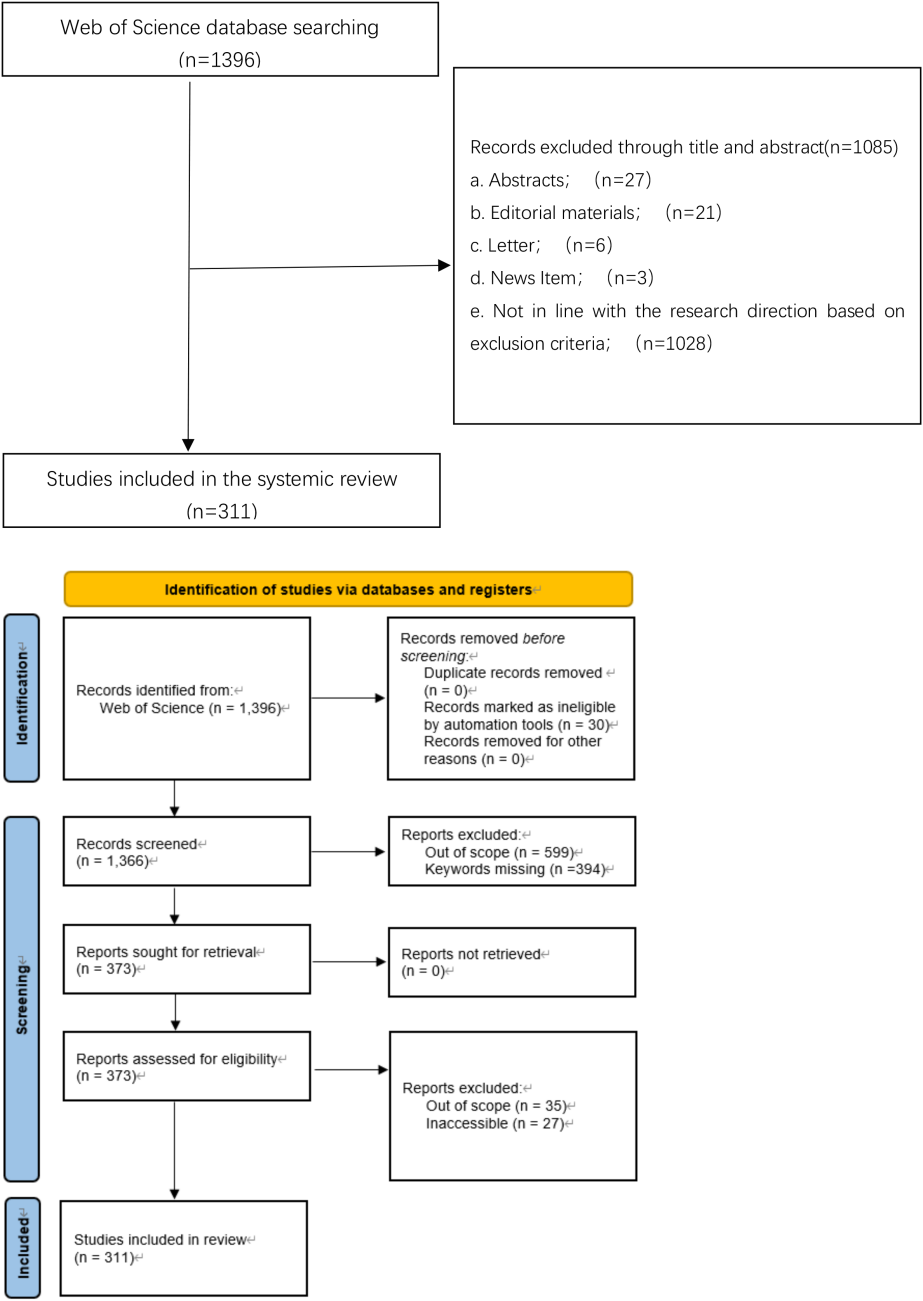
Up-screen flow chart; down-PRISMA flow diagram of the literature selection process.

The initial search in the Web of Science Core Collection database (WOSCC)identified a total of 1,396 documents based on the specified search strategy. Following a rigorous screening process, several documents were excluded: 27 abstracts, 21 editorial materials, 6 letters, 3 news reports, and 1028 documents that did not align with the research focus on rVSV vector vaccines. Ultimately, 311 papers met the inclusion criteria and were selected for future analysis. The detailed screen process is illustrated in the flowchart (**Figure 1**).

#### 3.1.1 Detailed screening and exclusion criteria

A two-step screening strategy was applied in the exclusion step (Step e, **Figure 1**) to ensure that retrieved documents aligned with the research focus on recombinant vesicular stomatitis virus (rVSV) vector vaccines. First, articles were assessed based on their Web of Science subject categories, and only publications within relevant disciplines were retained, including *Immunology*, *Medicine Research & Experimental*, *Virology*, *Microbiology*, *Biotechnology & Applied Microbiology*, *Pharmacology & Pharmacy*, *Biochemical Research Methods*, and *Oncology*. Second, the content of each article was manually examined to determine whether it explicitly involved the application of rVSV as a **vaccine or therapeutic agent**. Publications that focused solely on the biology, ecology, surveillance, or epidemiology of vesicular stomatitis virus (VSV), without addressing rVSV-based vaccine or therapeutic development, were excluded.

Representative examples of excluded studies include *“Surveillance along the Rio Grande during the 2020 Vesicular Stomatitis Outbreak Reveals Spatio-Temporal Dynamics of Viral RNA Detection in Black Flies”* and *“Detection of Vesicular Stomatitis Virus Indiana from Insects Collected during the 2020 Outbreak in Kansas, USA”*, which primarily addressed viral surveillance and outbreak epidemiology rather than rVSV vector applications. In addition, biological studies such as *“Zinc Finger Protein Designed to Target 2-Long Terminal Repeat Junctions Interferes with Human Immunodeficiency Virus Integration”* were excluded because they contained limited or no discussion of rVSV-based vaccine or therapeutic use.

#### 3.1.2 Measures to minimize selection bias

To minimize potential selection bias during the exclusion process, all articles at Step e were independently screened by three independent reviewers using the predefined exclusion criteria described in Section 1.6. Discrepancies in article inclusion or exclusion were resolved through group discussion and consensus.

Records were identified from the Web of Science Core Collection and screened by title and abstract. Records were excluded based on publication type or lack of relevance to rVSV vector vaccine applications according to predefined exclusion criteria. The remaining studies were included in the perspective.

### 3.2 Analysis results

#### 3.2.1 Publication volume trend

**Figure 2:**
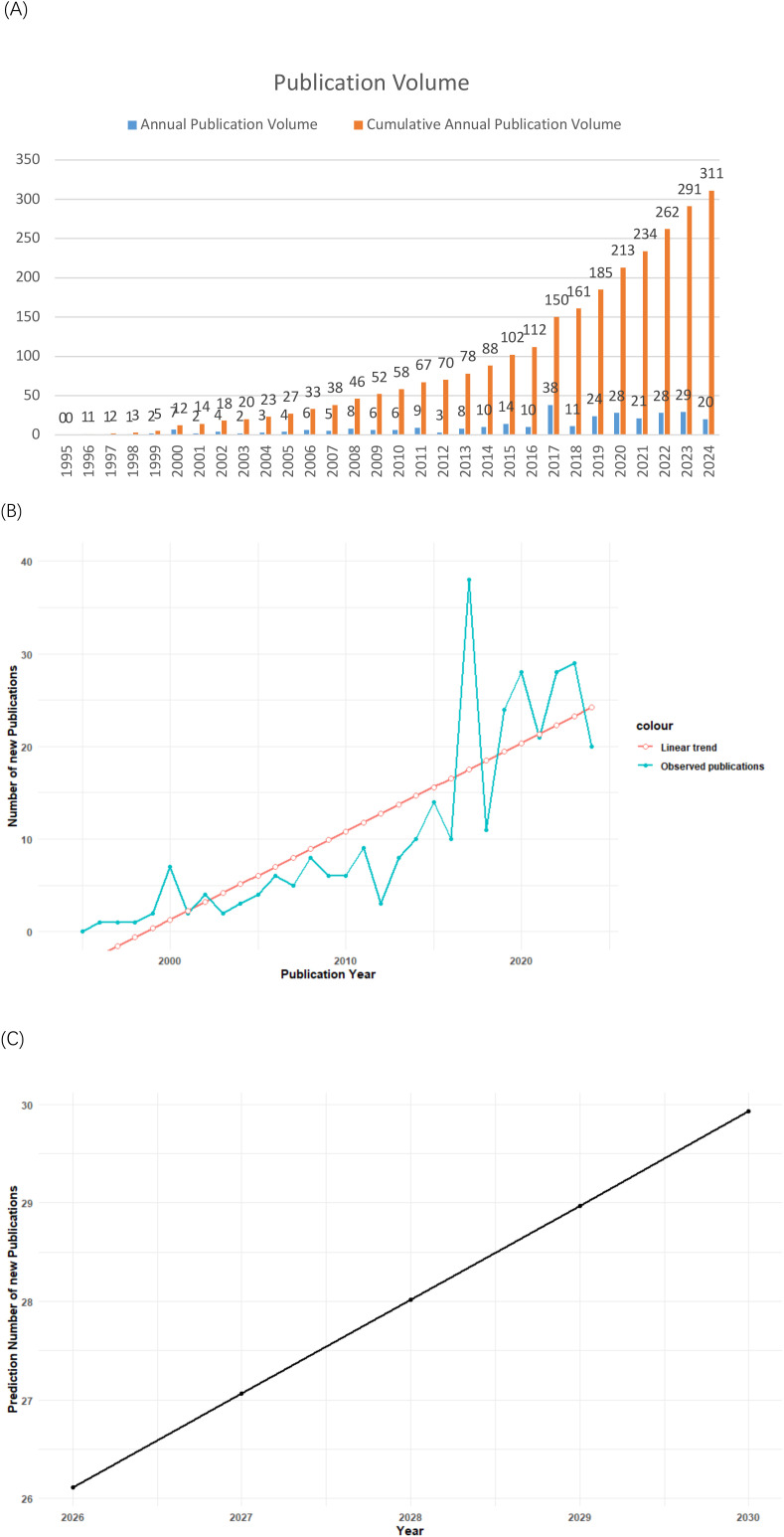
**(A)** Trend of recombinant VSV publication volume. **(B)** A linear regression model showing the number of articles published and the number of articles that were predicted to be published in the last 20 years on rVSV vector agents. **(C)** Linear regression model for predicting the number of publications in the field of rVSV vector agents.

From 1996 to 2024, a total of 311 publications related to rVSV were identified. The trend analysis reveals a steady increase in research activity, with a notable peak in 2017, indicating a growing interest in rVSV technologies over time (**Figure 2A**). The cumulative annual publication volume shows a consistent upward trajectory since then on rVSV-based applications in both vaccine development and immunotherapy.

In line with the approach demonstrated in the referenced study (10), Linear regression analysis was performed using R to evaluate the temporal trend in annual publication output. As shown in **Figure 2A**, the number of publications exhibited an overall increasing trend over time, despite noticeable year-to-year fluctuations in certain periods.

The linear regression model (**Figure 2B**) revealed a statistically significant positive association between publication year and annual publication count (β = 0.95 publications per year, *p* = 2.32 × 10^-8^), indicating a sustained growth in research output related to rVSV vector vaccines. The model explained a substantial proportion of the variance in annual publication numbers (R² = 0.68), suggesting a reasonably good fit.

Based on the fitted regression equation, the annual number of publications for the next five years (2026–2030) was projected (**Figure 2C**). The predicted values suggest that research activity in the field of rVSV vector agents is likely to continue increasing in the near future. Although projections derived from a linear regression model may deviate from actual future publication counts due to inherent uncertainties and external influences, they nonetheless reflect the growing and sustained research interest in rVSV-based platforms.

#### 3.2.2 Analysis of authors’ collaboration network

The author collaboration analysis identified key contributors in the file. **Table 2** shows top-ranked authors based on citation frequency, with Heinz Feldmann leading, followed by John K Rose and Andrea Marzi. These authors have significantly contributed to the advancements of rVSV research, particularly in vaccine and oncolytic virus development.

**Table 2:** Top authors by citation count in recombinant VSV research.

Rank	Citations	Author
1	35	Feldmann, Heinz
2	29	Rose, JK
3	26	Marzi, Andrea
4	24	Geisbert, Thomas W
5	16	Feldmann, Friederike
6	14	Geisbert, Joan B
7	11	Agans, Krystle N
8	10	Mire, Chad E
9	9	Fenton, Karla A
10	8	Huttner, Angela

**Figure 3:**
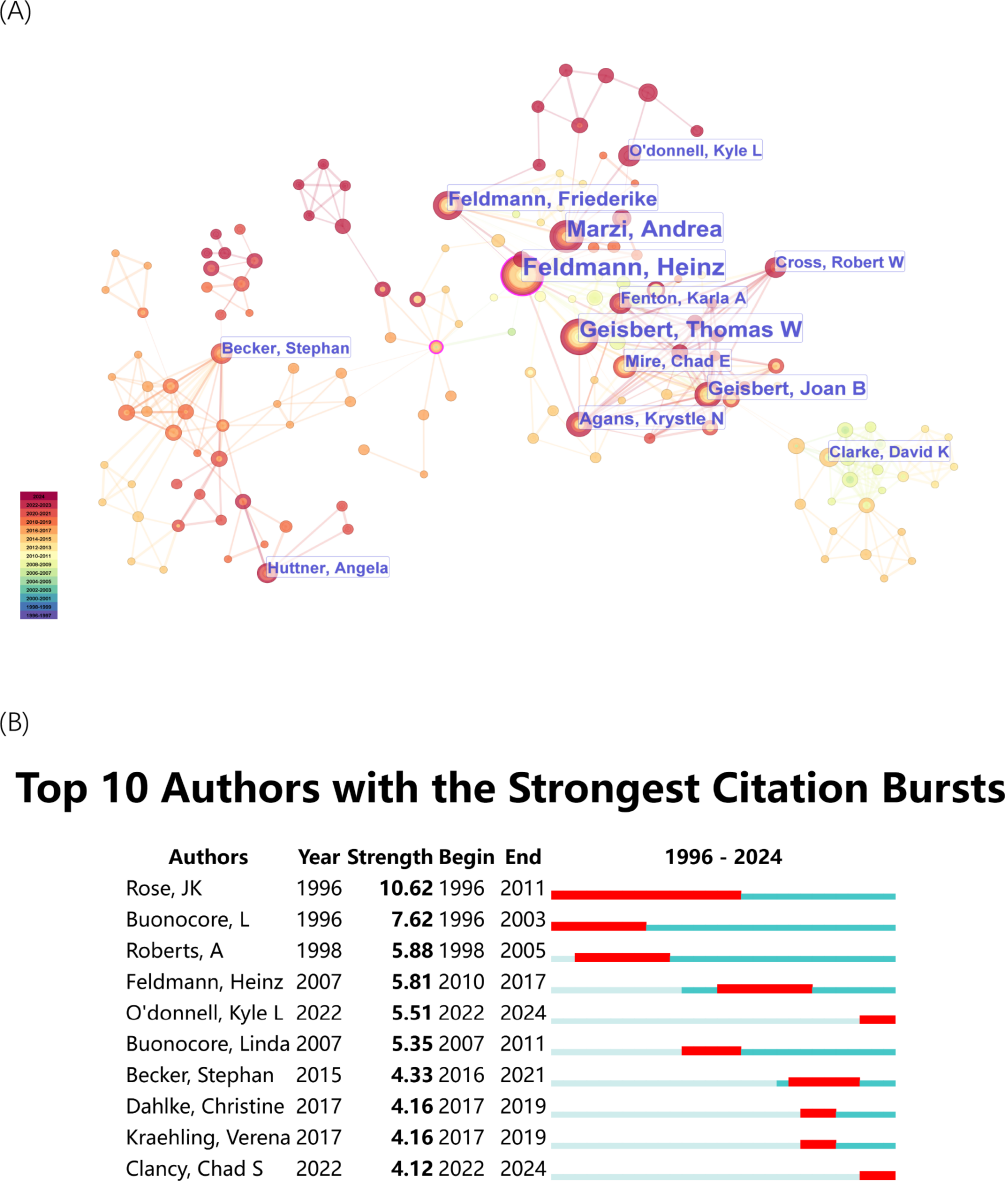
Authors’ co-citation analysis by Cite Space. **(A)** Co-citation Network of Authors. Dots represent authors, with larger dots indicating a high number of publications. Clusters are marked using different colors, and links represent cooperation between related authors. **(B)** Top 10 authors with the strongest citation bursts. The blue bar represents the timeline; the red bar represents the burst time period of the references, indicating the start year, end year, and duration of the outbreak.

The author co-citation analysis (**Figure 3**) illustrates the collaborative relationships among important researchers who work in rVSV research. The edges between nodes represent co-citation connections **Figure 3A**, highlighting prominent authors such as Heinz Feldmann, John K. Rose., Andrea Marzi and Thomas W. Geisbert. The clusters, indicated by distinct colors, represent groups of researchers working within specific subfields or research communities. The blue cluster in the network contains Rose Jk who has his key publications concentrated before 2010. His central position in the network demonstrates his leading role in the development of rVSV vaccines during the early stages of this research domain. The timeline at the bottom of **Figure 3A** extends from 1996 to 2024 to show how the number of authors and their connections between them grew denser after 2010. The research field of recombinant VSV studies shows increasing collaboration efforts and rising research activity according to the data. The authors with the most significant citation bursts appear in **Figure 3B** to demonstrate their academic influence during particular time periods. The research domain received its foundation from John K. Rose through his work which produced a citation burst from 1996 to 2011. Linda B. and Anjeanette Roberts demonstrate strong bursts which show their influence during particular time periods. The rVSV vaccine against Ebola virus received substantial support from Heinz Feldmann through his work from 2010 to 2017. The citation bursts of Kyle L. O’Donnell and Chad S. Clancy (both 2022-2024) demonstrate their increasing impact on current rVSV vaccine research developments. The citation bursts demonstrated how academic influence evolved dynamically because new researchers contributed to advanced research in the field.

#### 3.2.3 Analysis of institutional collaboration network

Between 1996 and 2024, the institutions with the highest impact on rVSV research include the National Institutes of Health (NIH), the NIH National Institute of Allergy & Infectious Diseases (NIAID), Yale University, University of Texas System, etc. (See **Table 3**).

**Table 3:** Institutions with a high number of citations.

No.	Citations	Institution
1	65	National Institutes of Health (NIH) - USA
2	58	NIH National Institute of Allergy & Infectious Diseases (NIAID)
3	33	Yale University
4	31	University of Texas System
5	30	University of Texas Medical Branch Galveston
6	21	Merck & Company
7	18	Public Health Agency of Canada
8	15	University of Manitoba
9	13	Philipps University Marburg
10	13	German Center for Infection Research

*. NIAID is a component of NIH. The identification of institutions and the analysis conducted is based on the affiliation labels in the papers.

**Figure 4:**
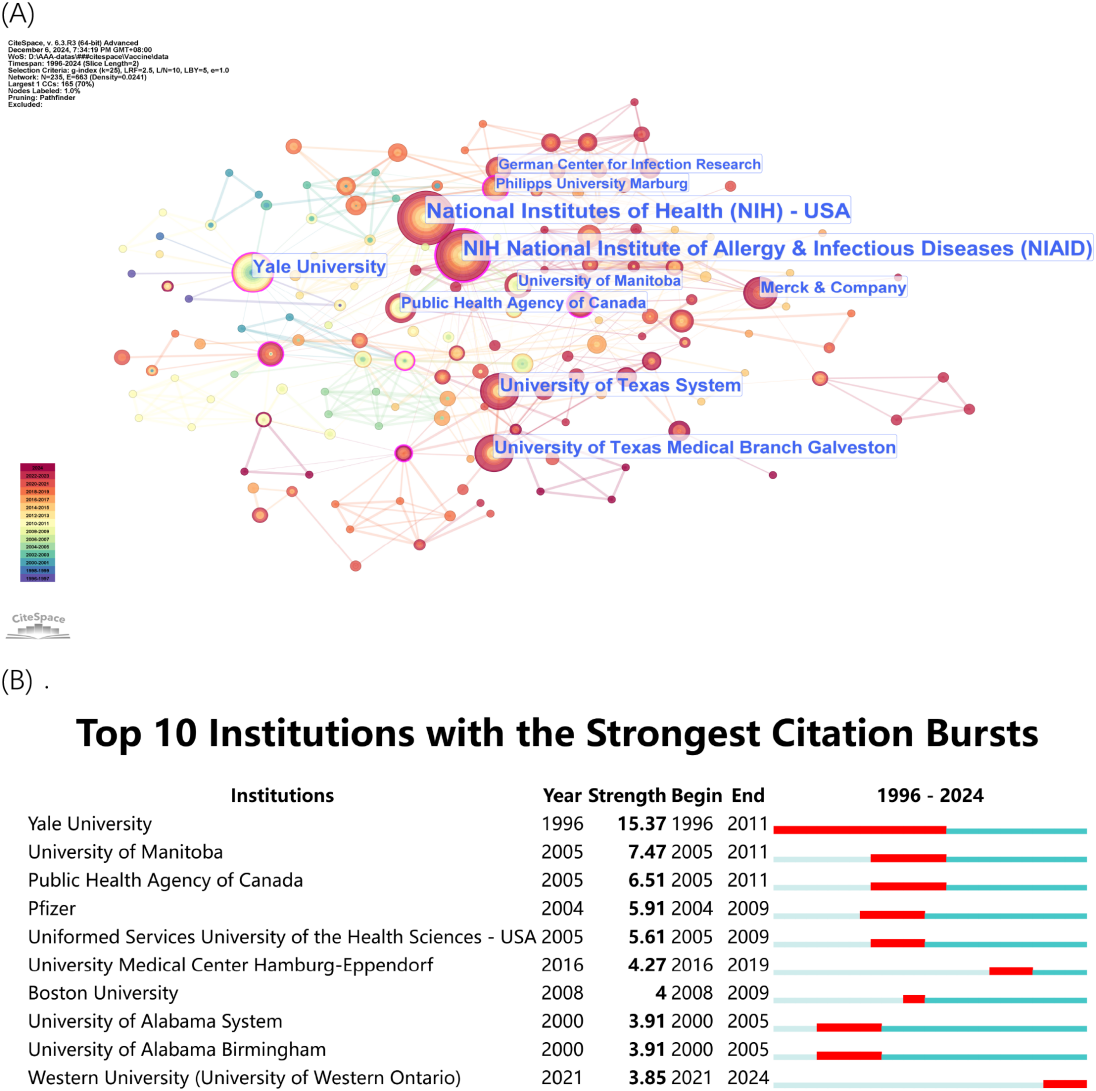
**(A)** Institutional Collaboration Network. Dots represent institutions, with larger dots indicating a higher number of publications and stronger collaborative ties. **(B)** Top 10 Institutions with the Strongest Citation Bursts. The timeline shows periods of significant citation increases, highlighting the institutions’ impact over time.

The institutional visualizations (**Figure 4**) presented the collaboration network (**Figure 4A**) which demonstrated the wide-ranging collaborative relationships between important research centers. The National Institutes of Health (NIH) and National Institute of Allergy & Infectious Diseases (NIAID) from the U.S. established strong partnerships between academic institutions and private sector entities. The research on rVSV vaccines received support from Merck & Company together with the Public Health Agency of Canada and various universities which demonstrated a multidisciplinary research approach. The institutions with the most significant citation bursts appear in **Figure 4B** which indicates periods of heightened research activity and impact. The research of John K. Rose at Yale University from 1996 to 2011 generated the most significant citation burst which established him as a leading figure in rVSV vaccine development. The University of Manitoba along with the Public Health Agency of Canada demonstrated significant citation bursts between 2005 and 2011 which corresponded to the essential time of rVSV vaccine development especially during the Ebola outbreak.

The research showed that U.S.-based institutions led global research initiatives but Canadian and European research centers made important contributions to the field. The collaboration between various institutions across different countries enabled the development of rVSV technology for therapeutic and preventive uses.

#### 3.2.4 Analysis of the international collaboration network

**Figure 5:**
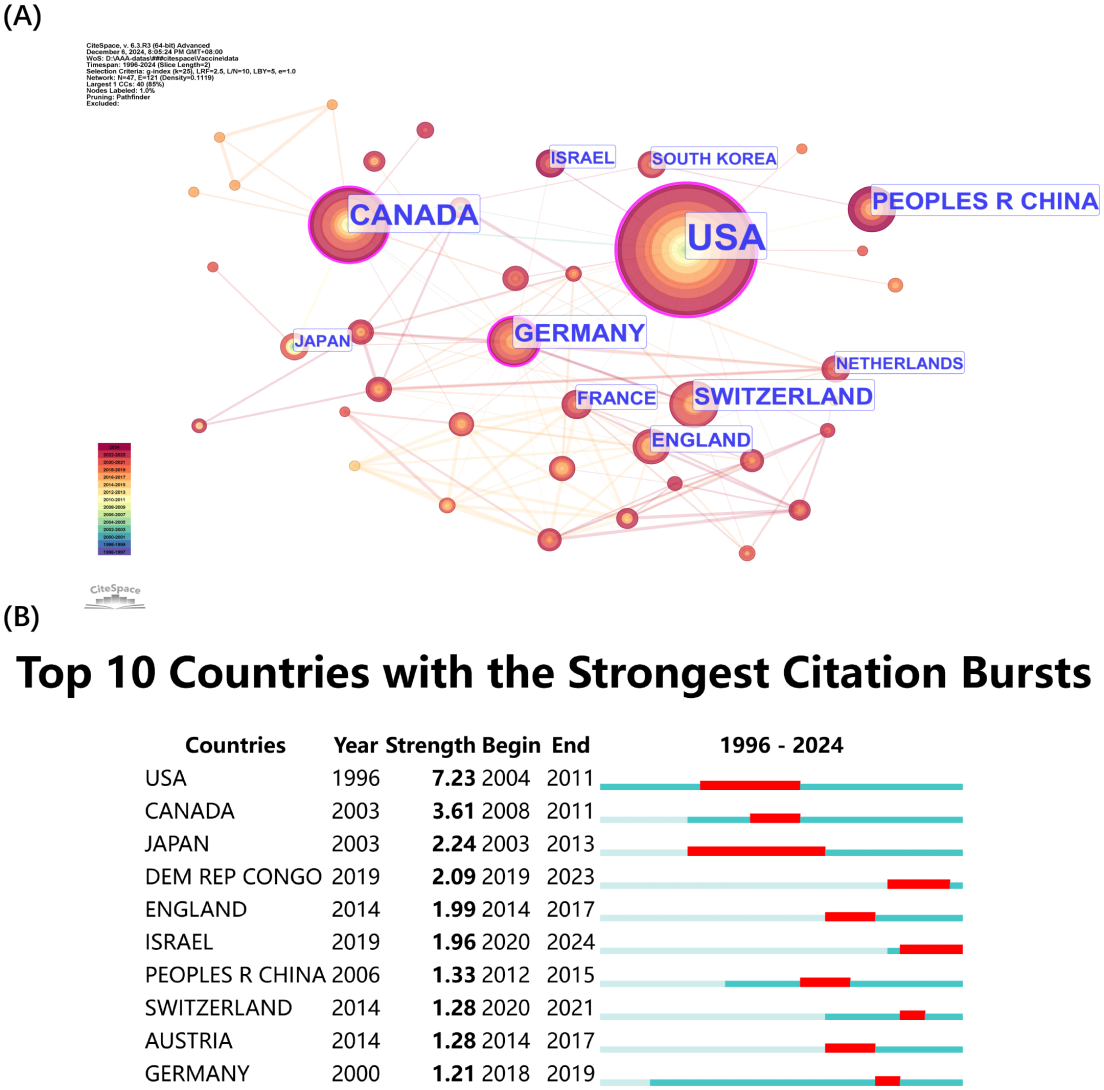
Countries co-citation analysis by Cite Space. **(A)** Co-citation Network of Countries. Dots represent countries, with larger dots indicating a high number of citations, clusters are marked using different colors and links represent cooperation between countries. **(B)** TOP 10 countries with the strongest citation bursts.

The network visualization presented in **Figure 5** displays citation volumes according to country (**Figure 5A**). The United States (USA) leads both citation volume and collaborative network (**Figure 5A**) because of its leading position in rVSV research advancement. The dense connections from the USA demonstrate its extensive global partnerships which demonstrate its worldwide research impact. The research field benefits from significant contributions of Canada and Germany through their strong citation counts. The research field shows extensive international participation through European countries Switzerland, England, and France and Asian contributors China and South Korea. The fourth position of China in the ranking demonstrates the nation’s expanding investments in vaccine development biotechnology. The top 10 countries with the strongest citation bursts are presented in **Figure 5B** which shows research activity peaks. The USA maintained the longest citation burst during 2004–2011 followed by Canada from 2008–2011 and Japan from 2003-2013. Several nations have displayed recent citation bursts including the Democratic Republic of Congo from 2019–2023 and England from 2014–2017 and Israel from 2020-2024. Research activities conducted by scientists from different continents including North America, Europe, Asia and Africa demonstrate the worldwide nature of research while confirming international collaboration plays a vital role in advancing rVSV vaccine research.

#### 3.2.5 Analysis of keyword cluster

The examination of keywords spanning from 1996 to 2024 shows essential research priorities within the field of rVSV. The top 10 most frequently cited keywords in **Table 4** demonstrate the main research directions of rVSV studies. The keyword “Vesicular stomatitis virus” stands as the most frequently mentioned term because it serves as the core subject of this field. The research focuses heavily on infection mechanisms and clinical trial designs and immune responses from vaccines because of the frequent appearance of ‘infection,’ ‘double-blind,’ and ‘immunogenicity’ keywords. The high number of citations for ‘nonhuman primates’ demonstrates their crucial role as testing models for vaccines. The research focuses on viral disease understanding and Ebola combat through the repeated use of terms including “safety,” “antibody,” “hemorrhagic fever,” “Ebola virus,” and “efficacy” to evaluate rVSV-based interventions’ safety and effectiveness.

**Table 4:** Top 10 most cited keywords in rVSV research.

No.	Citations	Keyword
1	80	vesicular stomatitis virus
2	77	infection
3	39	double blind
4	38	immunogenicity
5	36	nonhuman primates
6	34	safety
7	32	antibody
8	32	hemorrhagic fever
9	27	Ebola virus
10	27	efficacy

**Figure 6:**
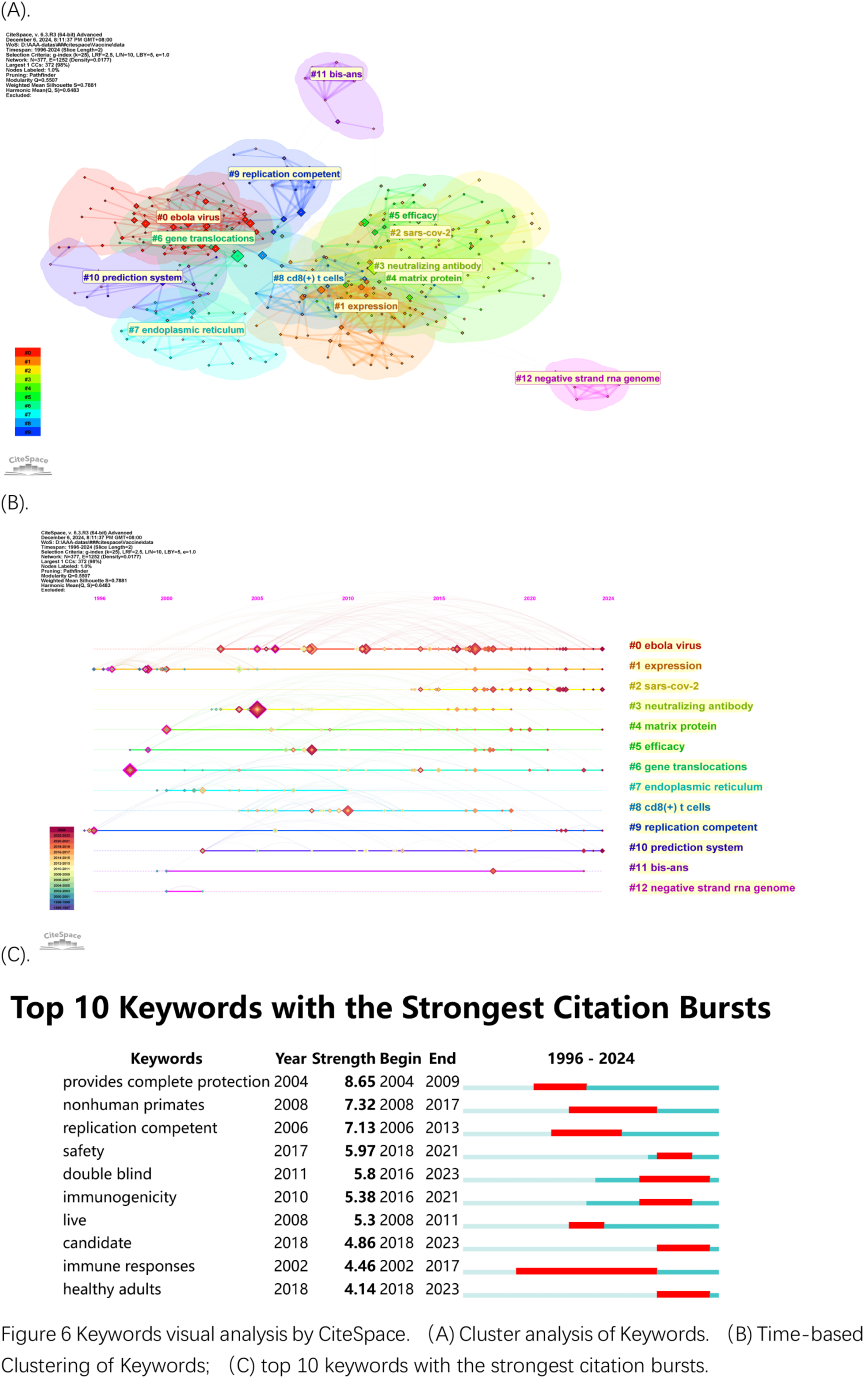
Keywords visual analysis by CiteSpace. **(A)** Cluster analysis of Keywords. **(B)** Time-based Clustering of Keywords; **(C)** top 10 keywords with the strongest citation bursts.

The network of co-occurring keywords in **Figure 6A** shows dense connections because research topics overlap extensively in this field. The largest cluster in **Figure 6A** has the label “Ebola virus” (#0). The keyword clusters numbered from 1 to 13 identified “Expression” as (#1) and “SARS-Cov-2” as (#2) and “neutralizing antibody” as (#3). Research has initially concentrated on using rVSV technology for vaccine development against Ebola before shifting to SARS-Cov-2 vaccine creation more recently. The presence of clusters focused on “glycoproteins” and “CD8+ T cell” response demonstrates research emphasis on both immune mechanisms and vaccine efficacy.

The following two figures examine time zone distributions (**Figure 6B**) and keyword bursts (**Figure 6C**). The high-frequency keyword development timeline is presented in the first figure but the second figure shows keyword bursts with their intensity and duration properties to indicate swift keyword transitions which represent new research directions. **Figure 6B** displays the temporal changes in keywords. The image contains small purple year labels at the top which match the colors of the years in the bottom left corner of the image. The visualization displays how research priorities shifted throughout time by tracking the appearance of specific keyword clusters which peaked during the Ebola virus (#0, red) and SARS-Cov-2 (#2) outbreaks with their respective terms “glycoproteins,” “neutralization antibody” (#3) and “protection” in the 2013–2016 Ebola epidemic and the 2019–2023 COVID-19 outbreak. The clusters #1 (dark red) for expression and #6 for endoplasm reticulum and #8 for CD8+ T cell and #12 (green) for negative strand RNA genome demonstrate ongoing research on viral replication and immune response mechanisms. The virus assembly research on Matrix protein (#4, brown) reached its highest point in 2015 and 2018. The research focus on vaccine development and protective immunity through rVSV studies persists throughout the entire period. The burst analysis in **Figure 6C** displays the ten most relevant keywords which represent periods of intense scientific investigation. Research has concentrated on testing rVSV vaccines for safety and effectiveness through “provides complete protection” and “nonhuman primates” and “replication-competent” and “safety” and “immunogenecity” and “double-blinded” and “immune responses” during health emergencies. The research bursts show how scientists have focused more intensely on rVSV applications when major outbreaks like Ebola and COVID-19 occur.

The keyword analysis confirms continuous interest in rVSV vaccine development particularly for addressing new infectious diseases as well as ongoing evaluation of immune responses and safety assessment. Research has adapted its direction to solve immediate global health issues through rapid development of rVSV-based vaccines.

#### 3.2.6 Analysis of reference clustering

Out of 311 studies on recombinant VSV vaccine development published between 1996 and 2024, **Table 5** lists the top 10 most frequently cited references, illustrating the foundational works that have significantly influenced the field. The most-cited reference, authored by Ana Maria Henao-Restrepo published in Lancet (11), undertook Ebola ça suffit! (translated as “Ebola that’s enough!”), a ring vaccination phase 3 efficacy trial in Guinea. The ring vaccination approach was inspired by the surveillance-containment strategy that led to smallpox eradication, and the primary objective of this trial was to assess the efficacy of the vaccine rVSV-ZEBOV efficacy of preventing Ebola virus disease in human beings. Preliminary results achieved 100% vaccine efficacy at the interim analysis. The next-ranked paper was written by J.A. Regules and J.H. Beigel, which published in the New England Journal of Medicine (12), detailed the outcomes of two phase 1 trials for an attenuated, replication-competent rVSV-based vaccine candidate (rVSVΔG-ZEBOV-GP) aimed at preventing Ebola virus disease.

**Table 5:** Top 10 most cited references in rVSV research.

No.	Citation	References
1	78	Henao-Restrepo AM, 2017, LANCET, V389, P505, DOI 10.1016/S0140-6736(16)32621-6
2	53	Regules JA, 2017, NEW ENGL J MED, V376, P330, DOI 10.1056/NEJMoa1414216
3	52	Agnandji ST, 2016, NEW ENGL J MED, V374, P1647, DOI 10.1056/NEJMoa1502924
4	44	Huttner A, 2015, LANCET INFECT DIS, V15, P1156, DOI 10.1016/S1473-3099(15)00154-1
5	26	Yahalom-Ronen Y, 2020, NAT COMMUN, V11, P0, DOI 10.1038/s41467-020-20228-7
6	25	Kennedy SB, 2017, NEW ENGL J MED, V377, P1438, DOI 10.1056/NEJMoa1614067
7	24	Heppner DG, 2017, LANCET INFECT DIS, V17, P854, DOI 10.1016/s1473-3099(17)30313-4
8	24	Suder E, 2018, HUM VACC IMMUNOTHER, V14, P2107, DOI 10.1080/21645515.2018.1473698
9	24	Marzi A, 2015, SCIENCE, V349, P739, DOI 10.1126/science. aab3920
10	21	Fathi A, 2019, HUM VACC IMMUNOTHERAPY, V15, P2269, DOI 10.1080/21645515.2019.1649532

**Figure 7:**
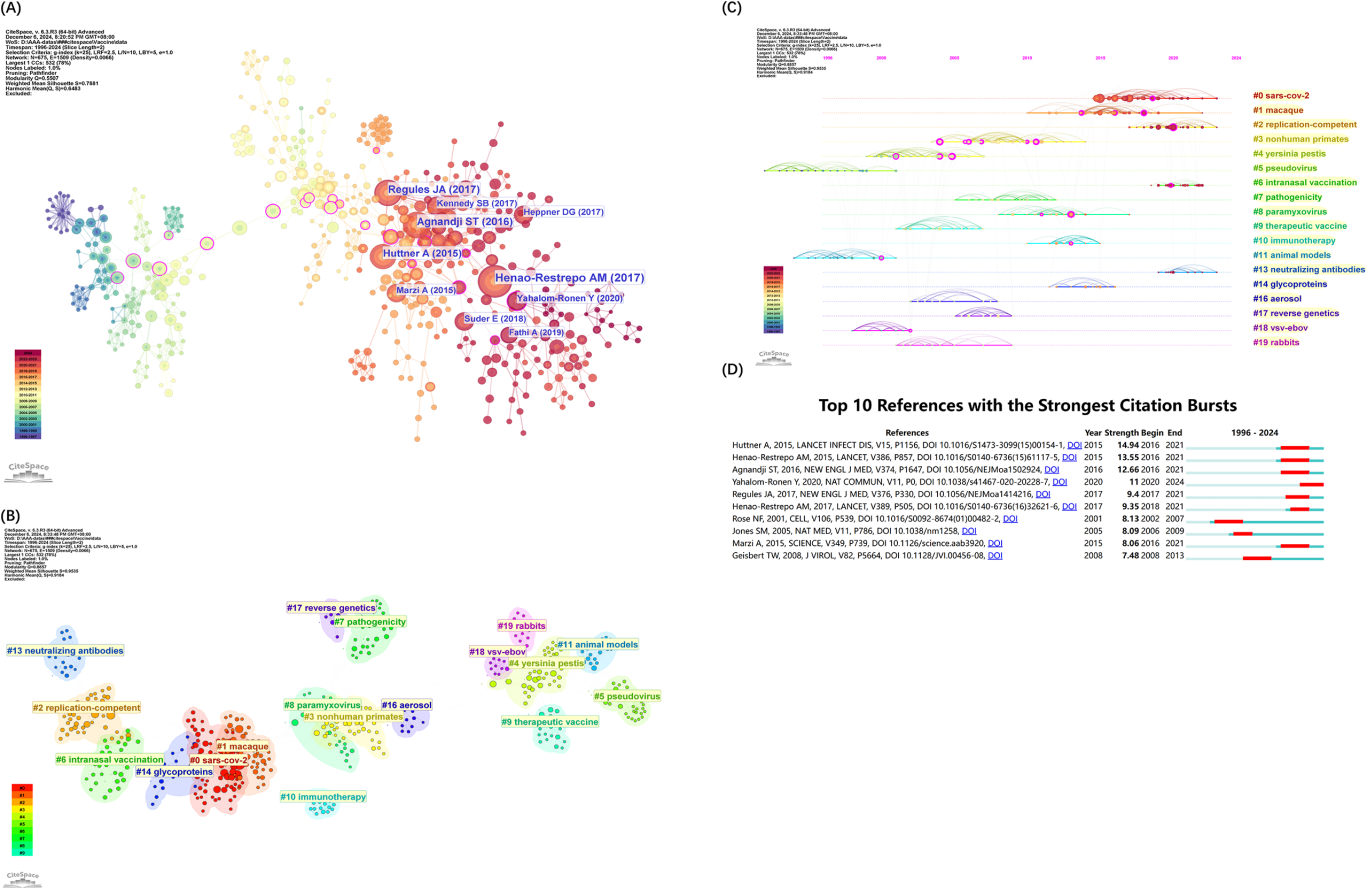
References visual analysis by CiteSpace. **(A)** References co-citation network. **(B)** Cluster view of co-citation of References. **(C)** Timeline Clustering of references. For each cluster, the location of each node represents when the literature was published, and the node size represents the number of citations. **(D)** The top 10 references with the strongest citation bursts. The blue bars represent the timeline, and the red bars indicate the start and end years of the burst duration.

Co-citation of references occurs when two or more papers are cited together by subsequent papers, establishing a co-citation relationship. **Figure 7A** illustrates the co-citation network of key references in rVSV vaccine studies, with each node representing an author and corresponding to the most-cited works listed in **Table 6**. The network’s connections highlight the collaborative nature and shared research focus among scholars. Using the Log-Likelihood Ratio (LLR) for clustering, the cited paper references are grouped into twenty labeled clusters, each characterized by corresponding keywords (#0-19) as shown in **Figure 7B**. The modularity Q value of 0.8857 suggests a well-defined network, and a silhouette score of 0.9535 confirms strong internal consistency of the clusters (13).

**Table 6:** Top 10 most cited articles in developing recombinant VSV vaccine research.

Rank	Author	Journal	Title	Citations of WoS	Institutions
1	Ana Maria Henao-Restrepo et al.	Lancet 2015;386(9996):857-66	Efficacy and effectiveness of an rVSV-vectored vaccine expressing Ebola surface glycoprotein: interim results from the Guinea ring vaccination cluster-randomized trial	562	World Health Organization, Geneva; University of Florida; London School of Hygiene & Tropical Medicine; etc.
2	Sean E Lawler et al.	JAMA Oncol 2017;3(6):841-849	Oncolytic Viruses in Cancer Treatment: A Review	365	Harvard Medical School, Boston, Massachusetts
3	Angela Huttner et al.	Lancet Infect Dis 2015;15(10):1156-1166	The effect of dose on the safety and immunogenicity of the VSV Ebola candidate vaccine: a randomized double-blind, placebo-controlled phase 1/2 trial	218	Geneva University Hospitals and Faculty of Medicine; Philipps University Marburg; World Health Organization, Geneva; etc.
4	James Brett Case et al.	Cell Host Microbe 2020;28(3):475-485.e5	Neutralizing Antibody and Soluble ACE2 Inhibition of a Replication-Competent VSV-SARS-CoV-2 and a Clinical Isolate of SARS-CoV-2	160	Washington University School of Medicine, St. Louis; The Donnelly Centre, University of Toronto; Vir Biotechnology, San Francisco, etc.
5	Thomas W Geisbert et al.	Vaccine 2008;26(52):6894-900	Vesicular stomatitis virus-based vaccines protect nonhuman primates against aerosol challenge with Ebola and Marburg viruses	157	Boston University School of Medicine, Boston, MA, USA
6	James Brett Case et al.	Cell Host Microbe 2020;28(3):465-474.e4	Replication-Competent Vesicular Stomatitis Virus Vaccine Vector Protects against SARS-CoV-2-Mediated Pathogenesis in Mice	108	Washington University School of Medicine, St. Louis, etc.
7	Steven M Jones et al.	J Infect Dis 2007;196 Suppl 2:S404-12	Assessment of a vesicular stomatitis virus-based vaccine by use of the mouse model of Ebola virus hemorrhagic fever	96	National Microbiology Laboratory, Public Health Agency of Canada, Canada
8	Anne Rechtien et al.	Cell Rep 2017;20(9):2251-2261	Systems Vaccinology Identifies an Early Innate Immune Signature as a Correlate of Antibody Responses to the Ebola Vaccine rVSV-ZEBOV	85	University Medical Center Hamburg-Eppendorf, Germany; Philipps University Marburg, Germany
9	Byram W Bridle et al.	Mol Ther 2009;17(10):1814-21	Vesicular Stomatitis Virus as a Novel Cancer Vaccine Vector to Prime Antitumor Immunity Amenable to Rapid Boosting with Adenovirus	83	McMaster University, Hamilton, Ontario, Canada
10	Yuxiao Wang et al.	Hum Vaccin Immunotherapy 2017;13(1):153-168	Ebola vaccines in clinical trial: The promising candidates	76	School of Public Health; Southeast University, Nanjing, PR China

From the co-citation data, keywords were mined for noun phrases, clusters annotated, and a timeline diagram of co-cited works was generated (**Figure 7C**). The node’s position from left to right signifies the publication year of the cited articles, illustrating their temporal distribution and the evolution of research themes (Wang et al., 2021). Notably, active clusters such as “SARS-CoV-2” (#0), “replication competent” (#2), intranasal vaccination (#6) and “glycoprotein” (#13) have emerged as enduring research hotspots from 2023 through 2024.

Burst references are those that have been widely cited during a specific period, indicating significant attention. **Figure 7D** shows the top 10 strongest citation bursts of references between 1996 and 2024. The first citation burst occurred in 2002, marked by a publication from Rose NH (14), which reported the development of an AIDS vaccine based on attenuated VSV vectors expressing env and gag genes, tested in rhesus monkeys. This paper, authored by John K. Rose, highlights his creative and significant influence in this area.

There are also two articles published in the past five years that have long-lasting, strongest citation-bursts through 2024. Yahalom-Ronen Y’s article, with the citation burst strength 10.98, reported that a single dose of the recombinant VSV-ΔG-spike vaccine provides protection against SARS-CoV-2 challenge in a golden Syrian hamster model. This demonstrates that the recombinant VSV-ΔG-spike could be developed as a safe, efficacious, and protective vaccine against SARS-CoV-2 (15). Remarkably, this preclinical success led to the initiation of a human clinical trial, phase I/II trial registered under the number NCT04608305(**Table 7**), phase II/III under the number NCT04990466. Unfortunately, although both preclinical and phase 1/2 trials have demonstrated no safety signals of concern and have further demonstrated immunologic response that approximates the response seen in convalescent individuals, the phase II/III trial was not able to reach completion. It was withdrawn on February 28, 2022, with the reason listed on ClinicalTrials.gov as lack of government funding, rather than inferiority to the controlled vaccine, which underscores the complexities of advancing promising candidates from bench to bedside (16). The other study, authored by Fathi A in 2019, provided a detailed analysis of vaccine platforms aligned with WHO’s Blueprint on emerging priority pathogens (17). With vesicular stomatitis virus (VSV) established as a reliable vaccine vector for over a decade, recent VSV-EBOV trials against Ebola have contributed significant clinical data, reinforcing VSV’s potential as a vector for outbreak pathogens. This review explores findings from both VSV-EBOV clinical trials and animal studies on vaccine candidates for Blueprint pathogens.

**Table 7:** Clinical trials of rVSV-based vaccines for infectious diseases (searched on Dec 24, 2025, with primary completion date on/after January 1, 2019).

Trial ID	Involving biological	Trial name	Condition	Phase	Primary completion	Status and results
NCT04608305	rVSV-SARS-CoV-2-S Vaccine	Evaluate the Safety, Immuno-genicity and Potential Efficacy of an rVSV-SARS-CoV-2-S Vaccine	Covid- 19	I/II	10/3/2022	completed
NCT03031912	V920 (rVSVΔG-ZEBOV-GP)	African-Canadian Study of HIV-Infected Adults and a Vaccine for Ebola - ACHIV-Ebola	Ebola (Zaire)	II	10/20/2024	active_not_recruiting
NCT05724472	rVSVΔG-SEBOV-GP Vaccine	Evaluation of Safety and Immunogenicity of rVSVΔG-SEBOV-GP Vaccine in Adults With Good General Health	Ebola (Sudan Virus)	I	1/9/2024	completed
NCT05909358	rVSV-SUDV CAd3; ChAdox1	Solidarity/Tokomeza Ebola Trial	Ebola (Sudan) Virus	I/II	2027-09	not_yet_recruiting
NCT05959421	VSV-EBOV	Immunity Induced by VSV-EBOV and Assessment of a Booster Dose in Individuals at Potential Occupational Risk for Exposure	Ebola	III	2027-12	not_yet_recruiting
NCT02876328	Ebola vaccine	Partnership for Research on Ebola Vaccinations	Ebola	II	12/24/2019	completed
NCT05130398	rVSVΔG-ZEBOV-GP Ebola Vaccine	Safety and Immunogenicity of the rVSVΔG-ZEBOV-GP Ebola Virus Vaccine Candidate in Children Living in Lambaréné, Gabon	Ebola	I/II	9/8/2021	completed
NCT04906629	INO-4201	INO-4201 as Booster in Healthy VSV-ZEBOV Vaccinees	Ebola	I	1/5/2022	completed
NCT06100913	Ebola Vaccine	Immunology of Ebola Vaccine	Ebola	II	8/31/2026	recruiting
NCT06841614	Ebola Mab/Vaccine	Ebola Post-Exposure Prophylaxis	Ebola	III	8/1/2027	not_yet_recruiting
NCT06587503	rVSVΔG-ZEBOV-GP + mRNA COVID	Safety and Immunogenicity of rVSVΔG-ZEBOV-GP Vaccination When Dosed Concurrently With mRNA COVID-19 Vaccine Booster Doses	Ebola |COVID-19	IV	2024-12	not_yet_recruiting
NCT02788227	rVSVΔG-ZEBOV-GP	Immunogenicity of Recombinant Vesicular Stomatitis Vaccine for Ebola-Zaire for Pre-Exposure Prophylaxis (PREP)	Ebola-Zaire	II	6/17/2025	completed
NCT05868733	Lassa Fever Vaccine	A Lassa Fever Vaccine Trial in Adults and Children Residing in West Africa	Lassa Fever	II	2026-12	recruiting
NCT04794218	rVSV-LASV-GPC Vaccine	A Clinical Trial to Evaluate the Safety and Immunogenicity of rVSVΔG-LASV-GPC Vaccine in Adults in Good General Health	Lassa Fever|Lassa Virus	I	12/19/2023	active_not_recruiting
NCT06265012	rVSV-Marburg Virus Vaccine	Study to Evaluate the Recombinant VSV (rVSV)-Marburg Virus Vaccine Candidate (PHV01)	Marburg Virus	I	9/23/2024	completed
NCT05178901	RVSV-Nipah PHV02)	A Phase 1 Study to Evaluate Safety & Immunogenicity of RVSV-Nipah Virus Vaccine Candidate PHV02	Nipah Virus	I	5/30/2023	completed
NCT06221813	rVSV-Nipah Virus Vaccine (PHV02)	Study to Evaluate Safety and Immunogenicity of a Prime-Boost Regimen of rVSV-Nipah Virus Vaccine Candidate PHV02	Nipah Virus	I	9/30/2024	completed

These findings underscore the dynamic nature of rVSV research, with historical focus on Ebola virus vaccines now expanding to include SARS-CoV-2 and other new emerging infectious diseases. The co-citation and burst analyses highlight key studies that have shaped the development and deployment of rVSV-based vaccines, emphasizing their impact on public health and infectious disease control.

#### 3.2.7 Top 10 highly cited articles in rVSV research

Within the corpus of 311 studies on rVSV vaccine development, **Table 6** lists the top 10 most-cited articles. The leading article by Henao-Restrepo et al., published in Lancet, details the Guinea ring vaccination using an rVSV-vectored Ebola vaccine, highlighting its high efficacy and safety (11). The article occupying the second position in citations presents an exposition on the nascent utility of the rVSV vector vaccine, delineating the current landscape of oncolytic viruses in clinical trials (18). Notably, the tenth-ranked paper, from Southeast University, China, offers a thorough review of the development of Ebola vaccines, from the initial virus introduction to a variety of vaccine types based on their technical approach, up to current clinical trials, and discusses ongoing issues and challenges that need further investigation. This clear and well-structured review article understandably ranks among the top ten (19).

An analysis of the top 10 most cited articles reveals that their themes can be divided into three major modules, providing insights into the research trends and focal points in this field:

rVSV-Ebola Virus Research (6 articles): These studies primarily focus on evaluating the efficacy and safety of rVSV-based vaccines targeting Ebola virus (19). Studies include clinical trials such as the Guinea ring vaccination (10) and detailed assessments of vaccine safety and immune response (20). Additionally, the research extends to vaccine effectiveness against another related filoviruses like Marburg, and tested against an aerosol challenge of both filoviruses (21); explores the systemic immune signatures induced by the vaccines (22), underscoring the pivotal role of rVSV technologies in advancing Ebola virus prophylactics.
rVSV Oncolytic Virus for Cancer Treatment (2 articles): This category of articles examines the use of rVSV as an oncolytic virus in cancer therapy with a particular emphasis on the good exploration of using VSV/Ad heterologous vaccination to enhance the magnitude of immune responses. Other highlighted works examine the potential of rVSV vectors in oncolytic virotherapy, discussing how these vectors kill cancer cells and the advantages of using rVSV for quick boosting of antitumor immunity. These studies contribute to a better understanding of how oncolytic viruses can be engineered to improve cancer immunotherapy (18, 23, 24).
rVSV-SARS-CoV-2 related Research (2 articles): This section of research deals with the use of rVSV vectors to combat SARS-CoV-2, the virus responsible for COVID-19 (25, 26). Both findings were produced by the same group and are important for the development of creative vaccine strategies and therapeutic approaches to deal with the ongoing challenges posed by COVID-19.

The most cited papers in rVSV research demonstrate the diversity of rVSV vectors in addressing different global health issues. From the fast deployment of Ebola vaccines to exploring new cancer treatments and dealing with the COVID-19 pandemic, rVSV-based technologies have shown to be a strong platform with many uses. This diverse set of research shows the requirement for continuous innovation and collaboration to exploit rVSV vectors for both therapeutic and preventive interventions.

### 3.3 Recent advances in the development of rVSV vector-based agents

#### 3.3.1 Development of rVSVs vaccines for preventing emerging infectious diseases, along with corresponding clinical trials

As a vaccine vector, VSV has garnered significant attention due to its high immunogenicity. However, there’s rarely pre-existing immunity in human populations, so repeated administration is effective without interference by the pre-existing antibody (27, 28). VSV replicates exclusively in the cytosol and doesn’t integrate into the host genome, providing a favorable safety profile (29, 30). Beyond pre-exposure immunization, rVSV vaccines have shown pronounced efficacy in both prophylactic and therapeutic contexts, including life-saving post-exposure protection against Marburg virus (21) and substantial protection against Ebola virus (31). These findings highlight the platform’s clinical value in outbreak response scenarios where immediate intervention is critical. Despite these advancements,

To enhance immunogenicity, researchers have utilized the transcriptional activity of the VSV promoter by relocating essential viral genes to the 5’ end of the genome, by which the replication rates were reduced while immunogenicity was maintained. Furthermore, repositioning the glycoprotein gene at the 3’ end has been shown to improve immune responses in mice (32, 33). Despite these advancements, robust neutralizing antibody responses against the vector hinder re-administration (34), limiting its use in repeated outbreak waves. Moreover, potential neurotoxicity due to VSV’s neurotropism remains a pressing safety concern (35) that must be carefully monitored in clinical deployment.

In 2020, Munis coined the term “recombinant VSV vector-based vaccines,” which refers to the vaccine products, whose G protein of wild-type VSV is replaced with glycoproteins from other viruses, listing good examples for a range of diseases, including measles, influenza, Lassa, Ebola, SARS, MERS, Zika, and HIV-1 (36). However, earlier rVSV vaccines, such as the recombinant Andes virus (37, 38) and the Marburg virus vaccine (39), were not included in Munis’s list.

The rVSV platform achieved its most successful application through the FDA-approved Ebola vaccine rVSV-ZEBOV, which marked a major achievement in public health readiness (40, 41). The vaccine showed exceptional effectiveness in controlling Ebola virus disease during the 2014–2016 West Africa Ebola outbreak while offering immediate protection and decreasing disease spread. The ring vaccination strategies that used rVSV-ZEBOV proved essential for managing subsequent outbreaks, thus demonstrating their value in emergency response situations. The successful implementation of rVSV-based vaccines has led to the creation of additional vaccines that target multiple infectious diseases.

The rVSV vaccine development process continues through multiple completed or ongoing clinical trials, which aim to expand its disease applications and strengthen its robustness. The website https://clinicaltrials.gov/ provides the most current information about clinical trials in this field.

The clinical trials presented in **Table 7**, which searched with terms of “Virus Diseases | Other terms: VSV OR rVSVΔG OR rVSV | Vaccine | intervention studies “, with primary completion date on and after January 1, 2019, initially retrieved 25 trials. After manually screening by kicking out 5 items of abnormal status(unknown or withdrawn), 2 items of antitumor trials, and 1 non-VSV vector-related drug candidate, 17 trials remained. In addition to the previously discussed (in section 2.6)rVSV-SARS-CoV-2-S vaccine trial NCT04608305,these active trials demonstrated the increasing advancement of rVSV research in targeting Ebola(11 items), Lassa virus and Nipah virus diseases (2 items respectively), and 1 Marburg virus vaccine candidate.

Among 11 Ebola virus vaccine trials, 9 focused on the FDA and EU-approved rVSV-ZEBOV vaccine solely(such as aiming to evaluate long-term immune response in typical participants and immune response differences between HIV-positive adults and other groups, or examining different glycoprotein substitutions beyond the Zaire Ebola virus strain glycoprotein), while the other 2 items focused on Ebo(Sudan)virus, starting from June 2023(NCT05724472, NCT05909358). The Sudan ebolavirus (SUVD) and the Zaire ebolavirus are classified as different species, and vaccines and monoclonal antibodies that are effective against Zaire ebolavirus disease are unlikely to be of any use against SUVD. As for another high-fidelity species of Bundibugyo ebolavirus (BDBV), no clinical trials have been captured so far, showing its development remains at the preclinical and immunogenicity testing stages, with promising indications from animal models, but no human trials have been launched (42).

Two completed Phase 1 clinical trials (NCT05178901 and NCT06221813) began in 2022 focused on Nipah virus disease to evaluate the safety and immunogenicity of the recombinant VSV-Nipah virus vaccine candidate PHV02. PHV02 is a live, recombinant virus consisting of vesicular stomatitis virus (VSV; Indiana) with the gene for the Zaire ebolavirus glycoprotein (GP) (EBOV GP); in addition, the Nipah virus (NiV) G protein is also inserted and expressed. This dual-antigen design allows the rVSV vector to facilitate expression and immunogenic presentation of both EBOV and NiV surface glycoproteins, aiming to induce protective immunity against Nipah virus while leveraging the proven VSV vector platform. Noteably, a Phase 1 clinical trial (NCT06265012) evaluated the recombinant VSV (rVSV)-Marburg virus vaccine candidate PHV01 in healthy adult subjects. The trial, sponsored by Public Health Vaccines LLC. Given its naming convention, PHV01 is likely part of the same vaccine platform series as PHV02, which has been tested as an rVSV-based candidate against Nipah virus.

We also identified two clinical trials on Lassa fever, sponsored by the International AIDS Vaccine Initiative. A Phase 1 trial (NCT04794218) completed in 2023 assessed the preliminary safety and immune response of the rVSV-based Lassa virus glycoprotein vaccine (rVSVΔG-LASV-GPC). In parallel, a Phase 2 trial numbered NCT05868733, titled *“A Lassa Fever Vaccine Trial in Adults and Children Residing in West Africa,”* is currently ongoing and expected to be completed in December 2026. It aims to further evaluate the safety, immunogenicity, and potential efficacy of the candidate vaccine in a broader population, including pediatric participants in endemic regions.

In summary, the clinical trials show increasing interest in employing rVSV vectors to expand new vaccine candidates for viral diseases and to evaluate the robustness of immune response duration in various population groups. All the constructs utilize the recombinant vesicular stomatitis virus vector backbone, highlighting the adaptability of the rVSV platform for rapid development of vaccines targeting emerging viral threats.

#### 3.3.2 Development of rVSV as cancer therapeutics based on the oncolytic activity of VSV, along with clinical trial advancements

Therapeutic vaccination, a form of immunomodulation, plays a significant role in treating both infectious diseases and cancers. Oncolytic viruses, including those from families of herpes, adeno, and rhabdovirus, have been studied for their ability to selectively target and destroy cancer cells. Oncolytic virotherapy (OVT) utilizes viruses, not merely as delivery systems for transgenes, but also as active therapeutic agents targeting and lysing cancer cells. Unlike gene therapy, which focuses on delivering genetic materials, OVT uses the virus itself to destroy tumors. The first FDA-approved oncolytic virus therapy is a herpes simplex virus-based talimogene laherparepvec (T-VEC, also known as OncoVEX (GM-CSF)), and was approved on October 27, 2015 (43). This breakthrough spurred increased interest in utilizing cancer cells’ compromised antiviral defenses to enhance virus replication within tumors (44).

Several oncolytic virotherapies are now commercially available worldwide, including Rigvir (Riga virus), an unmodified ECHO-7 picornavirus; Oncorine (H101), an E1B-deleted recombinant adenovirus; and Teserpaturev/G47Δ (Delytact^®^), a third-generation, triple-mutated recombinant oncolytic herpes simplex virus type 1 developed by Daiichi Sankyo Co., Ltd. Teserpaturev/G47Δ is approved in Japan for treating malignant glioma and is also applied in clinical trials for prostate cancer, malignant pleural mesothelioma, and recurrent olfactory neuroblastoma (45).

VSV has emerged as a promising oncolytic agent due to its natural sensitivity to type I interferon (IFN) responses. VSV’s efficacy has been demonstrated in various cancer models, including cervical, breast, melanoma, and glioblastoma (46). Despite promising results in preclinical studies and early human trials, rVSV-based oncolytic vectors face significant challenges in clinical translation. The key obstacles include inconsistent efficacy in targeting cancer cells, even within tumors of the same tissue origin, and documented cases of VSV-induced encephalitis in both laboratory animals and humans (47) could also be seen. In solid tumors such as pancreatic cancer, therapeutic responses are inconsistent, largely due to microenvironmental barriers — including fibrosis, hypoxia, and high interstitial pressure — that restrict viral spread. These features mirror the real-world clinical challenges oncologists face when treating refractory tumors, underscoring the need for next-generation rVSV vectors with enhanced tumor penetration. The rapid clearance of VSV by the immune system, through mechanisms such as neutralizing antibodies and complement molecules (48), further diminishes rVSV’s appeal. These challenges have significantly constrained the anticancer potential of VSV, particularly in terms of delivering multiple doses to reduce tumor size and effectively targeting neoplastic cells.

To overcome these shortages, VSV has also been explored as an oncolytic vaccine often used alongside other agents to maximize tumor reduction (49). Recombinant VSV variants expressing tumor-specific antigens have also shown promise in strengthening anti-tumor immunity, particularly in melanoma and other cancers, by activating systemic T cell responses leading to tumor lysis (50, 51).

The clinical trials focusing on VSV involving tumor interventions presented in **Table 8**, searched with terms of “tumor OR cancer | Other terms: VSV OR rVSVΔG OR rVSV | treatment OR therapy | intervention studies), with “primary completion date” on and after January 1, 2019, initially retrieved 24 trials. After manually screening by kicking out 4 items of abnormal status(unknown, terminated or withdrawn), 10 for non-VSV vector-related candidate interventions, finally 10 trials remained.

**Table 8:** Clinical trials of rVSV-based cancer treatment (searched on Sep 4 2025, with completion date on/after January 1, 2019).

Trial ID	Involving biological	Trial name	Start date	Study completion	Phase	Status and results
NCT01628640	rVSV-hIFNβ	Viral Therapy in Treating Patients with Refractory Liver Cancer or Advanced Solid Tumors	2012-08-3	2019-04-19	I	Completed; No result posted.
NCT03017820	VSV-IFNβ-NIS	VSV-IFNβ-NIS in Treating Relapsed or Refractory Multiple Myeloma, Acute Myeloid Leukemia, or T-cell Lymphoma	2017-04-04	2032-04-01	I	Active; Recruiting
NCT02923466	VSV-IFNβ-NIS	Administration of VSV-IFNβ-NIS Monotherapy and in Combination with Avelumab in Pts With Refractory Solid Tumors	2017-04-17	2022-04-22	I	Completed; No result posted
NCT04046445	VSV-GP128	Phase 1b Study to Evaluate ATP128, VSV-GP128 and BI 754091 in Patients with Stage IV Colorectal Cancer	2019-07-22	2025-08-31	I	Active; Not recruiting
NCT03120624	VSV-IFNβ-NIS	VSV-IFNβ-NIS With or Without Ruxolitinib Phosphate in Treating Patients with Stage IV or Recurrent Endometrial Cancer	2017-09-15	2028-01-01	I	Active; Not recruiting
NCT03865212	VSV-IFNβ-TYRP1	Modified Virus VSV-IFNβ-TYRP1 in Treating Patients with Stage III-IV Melanoma	2019-06-12	2027-01-20	I	Active; Not recruiting
NCT03647163	VSV-IFNβ-NIS	Systemic VSV-IFNβ-NIS and Pembrolizumab in Refractory NSCLC and NEC	2019-04-09	2025-12-31	I/II	Active; Recruiting
NCT04291105	Voyager V1(VSV-IFNβ-NIS)	Phase 2 Trial of Voyager V1 in Combination with Cemiplimab in Cancer Patients	2020-4-24	2025-12-01	II	Active Recruiting
NCT05846516	VSV-GP154; Ezabenlimab; ATP150;ATP152	A Study to Evaluate ATP150/ATP152, VSV-GP154 and Ezabenlimab in Patients With KRAS G12D/G12V Mutated PDAC (KISIMA-02)	2023-03-13	2027-03-01	I	Active; Recruiting
NCT06508463	VSV-hIFNβ-NIS;Ipilimumab; cemiplimab	Intravenous Vesicular Stomatitis Virus in Patients With Peripheral T-cell Lymphoma	2024-01-05	2032-04-01	I	Active; Recruiting

Eight clinical trials out of the ten studies utilize rVSV-IFNβ (Vesicular Stomatitis Virus encoding human Interferon-β) as an oncolytic viral therapy, which unites VSV’s cancer-killing properties with IFNβ’s immune system activation. The therapy targets cancer cells for destruction and activates immune responses against tumors. The majority of rVSV constructs contain the Sodium Iodide Symporter (NIS) gene, which enables medical professionals to track virus spread throughout the body using radioactive iodine. These trials are not only exploring rVSV as a stand-alone virotherapy but also as a synergistic partner with immune checkpoint inhibitors, directly aligning rVSV with current oncology standards of care. This strategy underscores the translational goal: to transform rVSV from an experimental tool into a clinically relevant therapy for patients with advanced or refractory cancers. The rVSV-IFNβ-NIS virus targets Refractory liver cancer (NCT01628640) and Relapsed or refractory blood cancer (NCT03017820), as well as Endometrial Cancer (NCT03120624), Melanoma (NCT03865212), Refractory NSCLC (Non-Small Cell Lung Cancer), NEC (Neuroendocrine Carcinomas) (NCT03647163), and other Refractory Solid Tumors (NCT02923466, NCT04291105). All clinical trials remain active except NCT02923466, which finished but lacks published results.

The development of rVSV vectors for vaccine and cancer therapy demonstrates their ability to address various human health needs. The clinical translation of rVSV vectors faces multiple challenges, particularly in oncolytic therapy, yet these vectors demonstrate promise when used with immunomodulatory agents. For example, in a current KRAS-mutant PDAC trial, rVSV (VSV-GP154) is combined with protein vaccines (ATP150/152) and PD-1 blockade (Ezabenlimab) to boost tumor-specific immunity(See clinical trial NCT 05846516). The ongoing clinical trials work to overcome existing challenges while investigating fresh therapeutic applications, which position rVSV as a valuable weapon against infectious diseases and cancers.

## 4 Discussion

In this study, we analyzed 311 publications on rVSV research from the Web of Science database, spanning the years 1996 to 2024. Our analysis covered the most influential authors, institutions, and countries, and research trends in this field. The citation burst analysis (**Figure 3**) highlighted John Rose as a pioneering figure in the field, particularly for generating recombinant VSV expressing RSV glycoproteins, which demonstrates early potential as an RSV vaccine. This groundbreaking achievement was facilitated by his laboratory’s mastery of cloning and expressing foreign genes within recombinant VSV (52–54), which is not only a scientific advance but also a proof-of-concept that recombinant VSV could serve as a flexible vaccine backbone for clinically relevant pathogens.

From the institution and country-level analyses, it is evident that North American and European countries – especially the USA, Canada, and Germany - are at the forefront of rVSV research. Although China has invested significantly in this area, its contributions are yet to match the innovative outputs and citation impact of its Western counterparts. This suggests that Chinese researchers have room to enhance their influence through more creative and high-impact studies. The geographical distribution of outputs underscores that translating rVSV research into tangible medical products requires not only strong laboratory capabilities but also coordinated regulatory and public health infrastructures. Our keyword clustering analysis, timeline, and burst analysis reveal that rVSV research has progressed in a clinically logical way. This was followed by preclinical tests in animal models, moving towards human trials to evaluate immune responses, then culminating in large-scale double-blinded clinical trials eventually.

The research into the top 10 most frequently cited studies between 2015 and 2018 shows that researchers focused heavily on rVSV-based Ebola vaccines since multiple critical clinical trials were performed during this period. These research studies show the thorough process that leads to FDA approval while establishing rVSV-Ebola vaccines as authorized biological products. Nature Communications published the ninth article during the COVID-19 pandemic through an Israeli institute that used the rVSV platform to replace VSV G with the SARS-CoV-2 spike protein (15). The rapid transition of Israeli researchers to address the COVID-19 pandemic illustrates rVSV’s unique *clinical agility* — a platform that can be swiftly re-engineered to respond to unforeseen viral threats, making it highly relevant for global pandemic preparedness.

The top ten most referenced studies reveal rVSV research has three main subfields: (1) Ebola vaccines and other filoviruses, (2) cancer therapy, and (3) SARS-CoV-2 adaptation. The majority of research investigates the safety and effectiveness of rVSV-Ebola vaccines because these products play a vital role in fighting Ebola, along with other filoviruses. Cancer-focused studies, while fewer, demonstrate the ability of rVSV to exploit tumor cell vulnerabilities and to act as an immunotherapy enhancer — though with slower clinical progress compared to vaccines. The studies show that rVSV technologies demonstrate dual clinical promises while providing important solutions to emergency infectious diseases countermeasures and next-generation cancer therapeutics.

The process of transferring rVSV research from laboratories to clinical practice enables scientists to transform laboratory findings into practical health advantages. Our analysis of ClinicalTrials.gov (clinical trials active on or after January 1, 2019) shows efficient clinical studies that use rVSV for developing preventive vaccines and cancer treatments.

Over the last half-decade, medical testing has pushed rVSV vaccines further into scientific reach. In addition to well-known efforts targeting Ebola - especially strains from Zaire and Sudan - researchers now explore similar approaches for Nipah, Lassa, and Marburg. Work ranges from early human trials to large-scale observational studies, checking how safe these vaccines are, whether they prompt strong responses, if earlier doses still work later, and whether protection lasts over time. In cancer therapy, the immunoregulatory biologic agent rVSV-hIFNbeta-NIS demonstrates a dual advantage: selective tumor cell destruction and real-time monitoring of viral biodistribution via the NIS gene. This represents a clinically significant innovation, as it simultaneously addresses therapeutic efficacy and patient safety. Moreover, trials combining rVSV with immune checkpoint inhibitors align this platform with standard-of-care oncology approaches, underscoring its translational potential in the clinic.

The therapeutic potential of VSV in cancer treatment faced challenges since it failed to precisely target cancer cells during oncolytic virotherapy, partly due to hostile tumor microenvironments (fibrosis, hypoxia, interstitial pressure) that block viral spread. Similarly, vector immunogenicity — which led to success in Ebola vaccines — poses a challenge in oncology, where repeated administration is often required. This dichotomy illustrates how the same biological properties can drive clinical utility in one domain while hindering another.

## 5 Conclusion

This study provides a comprehensive evaluation of the worldwide development of rVSV vector vaccines and therapeutics, integrating bibliometric analyses with active clinical trial data. Our research revealed that North American and European nations have become the leaders in rVSV research productivity while maintaining the highest level of influence since 2017. China and other regions are increasing their contributions, but their clinical impact remains to be fully realized. The analysis of keywords together with references and highly cited articles helped both evaluate past research developments and predict upcoming trends. The analysis of the top 10 highly cited references together with access to current active clinical trials on rVSV biological products provided direct insights into current research hotspots and promising therapeutic applications for rVSV.

Looking forward, the upcoming research on rVSV will concentrate on enhancing safety and efficacy profiles and broadening therapeutic indications while developing better delivery methods. The future development of rVSV-based therapeutics will advance through three main innovations which include combinatorial therapies with immune checkpoint inhibitors and vector design improvements for host immunity evasion and targeted tumor site delivery. Overall, the trajectory of rVSV research illustrates how genetic engineering and translational science can transform a laboratory vector into clinically meaningful interventions. With continued improvements in vector design, regulatory oversight, and integration into combinatorial regimens, rVSV platforms are poised to make lasting contributions to both infectious disease preparedness and cancer treatment.

## Data Availability

The original contributions presented in the study are included in the article/supplementary material. Further inquiries can be directed to the corresponding authors.
